# Comparative Transcriptome Analysis between Fertile and CMS Flower Buds in Wucai (*Brassica campestris* L.)

**DOI:** 10.1186/s12864-018-5331-4

**Published:** 2018-12-12

**Authors:** Guohu Chen, Xinyu Ye, Shengyun Zhang, Shidong Zhu, Lingyun Yuan, Jinfeng Hou, Chenggang Wang

**Affiliations:** 1 Vegetable Genetics and Breeding Laboratory, College of Horticulture, Anhui Agricultural University, Hefei, 230036 China; 2 Anhui Provincial Engineering Laboratory of Horticultural Crop Breeding, Hefei, 230036 China

**Keywords:** Wucai, Cytoplasmic male sterility, Differentially expressed genes, Transcriptome analysis, Tapetum, RNA-Seq

## Abstract

**Background:**

Wucai (*Brassica campestris* L. ssp. *chinensis* var. *rosularis* Tsen) is a variant of nonheading Chinese cabbage (*Brassica campestris* L.), which is one of the major vegetables in China. Cytoplasmic male sterility (CMS) has been used for Wucai breeding in recent years. However, the underlying molecular mechanism of Wucai CMS remains unclear. In this study, the phenotypic and cytological features of Wucai CMS were observed by anatomical analysis, and a comparative transcriptome analysis was carried out to identify genes related to male sterility using Illumina RNA sequencing technology (RNA-Seq).

**Results:**

Microscopic observation demonstrated that tapetum development was abnormal in the CMS line, which failed to produce fertile pollen. Bioinformatics analysis detected 4430 differentially expressed genes (DEGs) between the fertile and sterile flower buds. Gene Ontology (GO) and Kyoto Encyclopedia of Genes and Genomes (KEGG) pathway enrichment analyses were performed to better understand the functions of these DEGs. Among the DEGs, 35 genes (53 DEGS) were implicated in anther and pollen development, and 11 genes were involved in pollen cell wall formation and modification; most of these showed downregulated expression in sterile buds. In addition, several genes related to tapetum development (*A6*, *AMS, MS1*, *MYB39*, and *TSM1*) and a few genes annotated to flowering (*CO*, *AP3*, *VIN3*, *FLC*, *FT*, and *AGL*) were detected and confirmed by qRT-PCR as being expressed at the meiosis, tetrad, and uninucleate microspore stages, thus implying possible roles in specifying or determining the fate and development of the tapetum, male gametophyte and stamen. Moreover, the top four largest transcription factor families (MYB, bHLH, NAC and WRKY) were analyzed, and most showed reduced expression in sterile buds. These differentially expressed transcription factors might result in abortion of pollen development in Wucai.

**Conclusion:**

The present comparative transcriptome analysis suggested that many key genes and transcription factors involved in anther development show reduced gene expression patterns in the CMS line, which might contribute to male sterility in Wucai. This study provides valuable information for a better understanding of CMS molecular mechanisms and functional genome studies in Wucai.

**Electronic supplementary material:**

The online version of this article (10.1186/s12864-018-5331-4) contains supplementary material, which is available to authorized users.

## Background

Wucai (*Brassica campestris* L. ssp. *chinensis* var. *rosularis* Tsen) is a variant of nonheading Chinese cabbage (*Brassica campestris* L.), which is the most important species in the *Brassicaceae* family [1]. As an important autumn and winter vegetable crop, this crop is cultured widely in most parts of China, where it originated, especially in the Yangtze-Huaihe River Basin, and has become increasingly popular in other countries for its beautiful shape and significant levels of vitamins and minerals [2]. In recent years, cytoplasmic male sterility (CMS) has been used in some cultivated breeds [3] to generate stronger plants and higher hybrid seed yield [4, 5].

Owing to an interaction between mitochondrial and nuclear genes, the CMS phenotype fails to produce functional anthers, pollen or male gametes [6]. Thus, understanding the delicate and complex processes of anther and pollen development is a prerequisite for comprehension of this unique phenomenon in CMS plants [5]. However, anther and pollen development is a critical phase in the plant life cycle, which contains a series of correlated events involving a diverse range of genes in complex regulatory networks [7–9]. Dysfunction of these genes may lead to male sterility [10]. Although many of these genes have been isolated and analyzed to have vital roles in CMS, the regulatory network and the novel genes underlying CMS occurrence are still largely unknown [8, 10].

In recent decades, genetic research into CMS occurrence has included two main types, map-based cloning and sequence-based transcriptome assays [7]. Using AFLP and SSR techniques for gene mapping, Xu et al. [11] identified the restorer gene *BrRfp* from the *pol*-like CMS restorer line of heading Chinese cabbage (*B. rapa*). Compared with the gene mapping method, the Illumina sequencing (RNA-Seq) technique could offer several key advantages over existing technologies [12]. This form of transcriptional analysis allows for the determination of genome-wide expression levels as well as identification of new genes and SNPs, especially genes with very low abundance [13–15]. Furthermore, the results of RNA-Seq also show high levels of reproducibility for both technical and biological replicates [16]. Therefore, taking these advantages into account, RNA-Seq has been used successfully in the pollen and anther development of *Brassica* crops, such as *B. napus* [6, 9, 14, 17], *B. rapa* [7, 18], *B. oleracea* [19–21], *B. campestris* [5], and *B. juncea* [22]. However, to the best of our knowledge, the genome-wide transcriptional profiles and related genes of fertile and sterile flower buds from Wucai have not yet been reported through RNA-Seq technology.

In our previous study, a newly sterile plant of Wucai was generated by hybridization with nonheading Chinese cabbage, and a stable sterility line was developed via backcrossing for ten generations. In this present study, the objective was to further understand the differences in the transcriptome between the CMS line and its maintainer line and to find some molecular clues to this CMS system. Accordingly, mRNA was isolated from the flower buds of fertile and sterile plants, respectively, and then, genome-wide transcriptional profiling was performed using the Illumina RNA-Seq platform. Based on bioinformatics analysis, a large number of candidate genes and transcription factors involved in anther and pollen development were isolated, and various screened candidate genes related to pollen development were further analyzed by qRT-PCR. Our results may contribute to an understanding of CMS molecular mechanisms and provide useful information for further heterosis breeding in Wucai.

## Results

### Phenotypic and cytological characterization

**Fig. 1:**
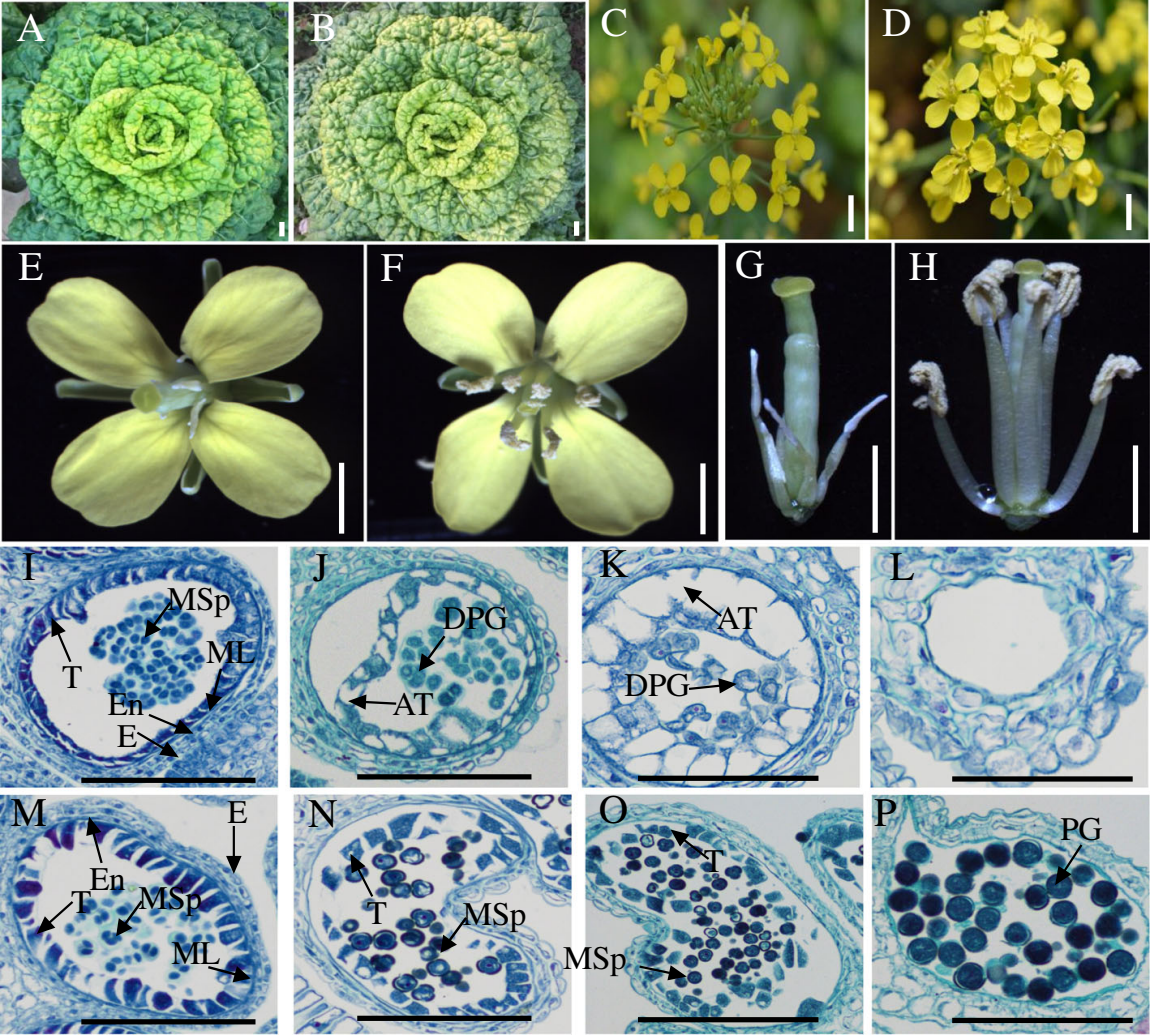
Morphological characteristics of flowers and microscopic observations of anthers from the sterile and fertile lines of Wucai. **a** and **b**, CMS line 12-14A and its maintainer line 12-14B. **c**, **e**, **g** and **d**, **f**, **h**, Inflorescences, flowers, and petals and stamens from the sterile and fertile lines, respectively. **i**-**l**, Transverse sections of sterile anthers; abnormal tapetum was formed, and the pollen sacs of sterile anthers did not produce pollen, in the CMS line. **m**-**p**, Transverse sections of fertile anthers; normal tapetum and mature anthers developed in the maintainer line. AT, abnormal tapetum; DPG, degenerated pollen grain; E, epidermis; En, endothecium; ML, middle layer; MSp, microspore; PG, pollen grain; T, tapetum. Scale bars in **a**, **b**, **c** and **d** represent 1 cm; scale bars in **e**, **f**, **g**, **h** represent 0.5 cm; scale bars in I-P represent 100 μm

After ten generations of backcrossing, there was no difference in morphological phenotype between the sterile line 12-14A and its maintainer line 12-14B (Fig. 1a and b), and the forms of the corolla and flower seemed normal (Fig. 1c-f). However, compared with those of the fertile flower, shorter filaments and undeveloped anthers were observed on the stamens of the sterile flower (Fig. 1g-h).

To accurately characterize the cause of the pollen abortion, semithin sections of the buds from the two lines of Wucai were observed. As shown in Fig. 1i and IM, there was no obvious difference in the meiosis period between sterile and fertile anthers. However, at the tetrad stage, the tapetal cells expanded, and the microspore could not carry out meiotic division (Fig. 1j). After this stage of anther development, the tapetal cells vacuolated and filled in the sacs, and the microspores degraded (Fig. 1k), which caused pollen abortion (Fig. 1l). In contrast, a normal tapetum and fertile pollen grains developed in the fertile anthers (Fig. 1m-p).

### De novo assembly and sequence annotation

To further understand the molecular mechanisms of CMS differences in Wucai, RNA-Seq was performed using Illumina technology. After the raw data were trimmed, a total of 52,936,673 clean reads for the fertile samples and 52,606,810 for the sterile samples were obtained, and the Q20 and Q30 were > 96.61 and > 92.53%, respectively (Table 1). In addition, the GC contents were consistently approximately 45% for both sterile and fertile samples (Table 1), suggesting that the sequencing was highly accurate. All clean reads (105,543,483) were assembled using the Trinity program [23]. As the result, 117,332 contigs were obtained with a mean length of 901 nt (Table 1). After clustering, 80,851 unigenes (> 200 bp) were generated; the average length was 1054 nt, and the N50 was 1586 nt (Table 1). The lengths of all unigenes were longer than 199 bp, and 86.95% of them ranged from 200 to 1999 bp (Additional file 1: Table S1). The assembled unigenes were subjected to search against the Nr, Swiss-Prot and COG databases, and 66,143 (81.81%), 54,857 (67.85%) and 28,129 (34.79%) unigenes were aligned against these three protein databases, respectively (Additional file 2: Table S2). The species distribution showed that that almost all of the sequences matched sequences from the *Brassicaceae* (Additional file 3: Figure S1).

**Table 1:** Illumina sequencing data and results of de novo assembly

	Sterile	Fertile	Total
Reads			
Clean reads	52,606,810	52,936,673	
Q20 (%)	96.61	97.29	
Q30 (%)	92.53	94.27	
GC content (%)	45.89	45.65	
Contig			
Total number			117,332
Total length (nt)			105,669,013
Mean length (nt)			901
N50 (nt)			1415
Unigene			
Total number			80,851
Total length (nt)			85,236,698
Mean length (nt)			1054
N50 (nt)			1586
Distinct clusters			43,191
Distinct singletons			37,660

### Identification of differentially expressed genes

**Fig. 2:**
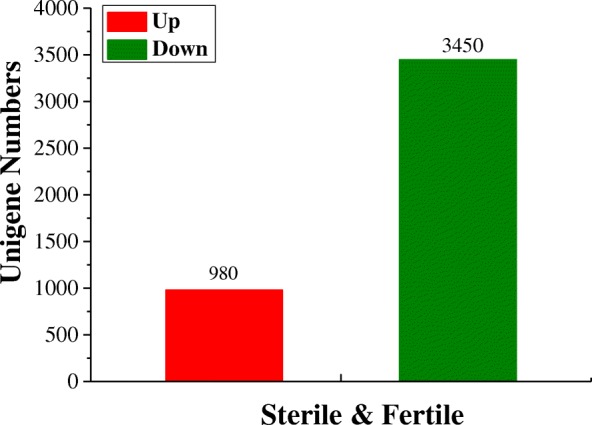
Number of DEGs between sterile and fertile buds. Red indicates upregulated DEGs, and green indicates downregulated DEGs

To gain better insight into the differences in gene expression patterns, we identified differentially expressed genes (DEGs) between the sterile and fertile lines. A total of 4430 genes (including 147 novel genes) were identified in the sterile and fertile comparison, including 980 genes upregulated and 3450 downregulated in sterile buds (Fig. 2; Additional file 4: Table S3). Among these DEGs, 1384 specifically expressed genes were observed that were expressed in only the fertile (1044) or sterile (340) samples. These results showed that the number of downregulated DEGs was considerably higher than that of upregulated DEGs. In addition, 147 novel genes were identified that were not annotated to any database. The biological functions of these novel genes remain to be determined (Additional file 5: Table S4).

### Functional annotation by Gene Ontology

To investigate the function of the DEGs, the genes that showed significant differential expression were subjected to analysis by Blast2GO software. As shown in Additional file 6: Figure S2, 4430 DEGs were categorized into 53 functional groups under three main classifications. Among these groups, ‘cellular process’ (2180; 49.21%) in biological process, ‘cell’ (2618; 59.10%) and ‘cell part’ (48,388; 87.59%) in cellular component, and ‘binding’ (1450; 32.73%) in molecular function were the dominant categories. Conversely, three categories (‘cell killing’, ‘virion’ and ‘metallochaperone activity’) had only a few unigenes.

### Pathway mapping by Kyoto Encyclopedia of Genes and Genomes

**Fig. 3:**
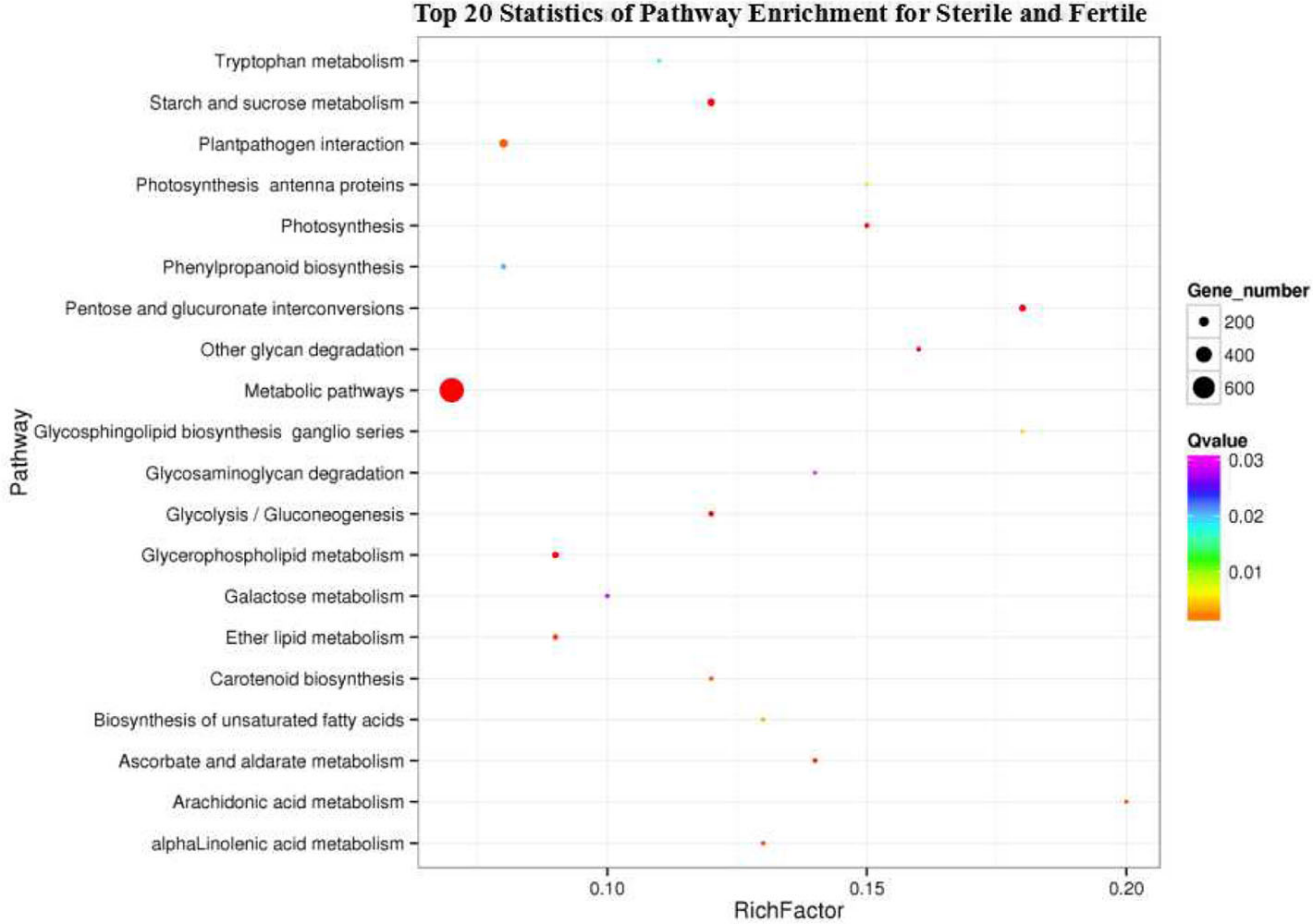
Twenty most significantly enriched KEGG pathways

To understand the biological functions of DEGs that might be active in Wucai, pathway annotation was performed against the Kyoto Encyclopedia of Genes and Genomes (KEGG) database. The results showed that 2217 of 4430 DEGs were assigned to 119 KEGG pathways (Additional file 7: Table S5). The 20 most significantly enriched KEGG pathways are shown in Fig. 3. The pathways with significantly more DEGs were metabolic pathways (676, 15.26%), biosynthesis of secondary metabolites (284, 6.41%), plant-pathogen interaction (162, 3.61%), and starch and sucrose metabolism (124, 2.80%). In starch and sucrose metabolism, a total of 124 DEGs were screened, and 38 of these DEGs were expressed in only fertile buds, while 71 DEGs were downregulated in sterile buds (Additional file 8: Table S6). These pathway annotations provide a basis for investigating gene functions involved in male sterility in Wucai.

### Genes related to anther and pollen development

Pollen development is a complex process that involves many events and plays an important role in plant propagation. In this study, all of the DEGs were annotated against the processes of anther and pollen development of *A. thaliana*. As shown in Table 2, 35 genes are considered to regulate male gametophyte development in Wucai. From *A6* to *ZAT5*, 30 genes were downregulated in sterile buds. In contrast, 5 other genes, *BT2*, *SCC12*, *TCMO*, *VAL2* and *XPO1*, were upregulated in sterile buds. Among these genes, *A6*, *AMS, ENL2*, *MS1*, *MYB39*, *ORTH2*, *PLRX1* and *TSM1* are also considered to be involved in tapetum development. In addition, we found several genes associated with cell wall formation and modification, such as the Pectinesterase gene (*PME5*), UDP-arabinose mutase gene (*RGP1*), and Cinnamoyl-CoA reductase gene (*CCR2*), which might participate in the processes leading to CMS in Wucai.

**Table 2:** Identities and relative expression of DEGs associated with anther and pollen development in Wucai

#	Gene name	Gene ID	log_2_ Ratio (S/F)	Up-Down- Regulation (S/F)	*P*-value	FDR	Nr-ID	Annotation	Note
1	A6	CL5312.Contig6	−2.944716046	Down	0.000000833	0.0000238	BAG16366.1	Tapetum-specific protein A6	Tapetum
2	ACA2	CL7501.Contig3	−22.02431	Down	1.32E-10	7.15E-09	AAF18608.2	Calcium-transporting ATPase 2, plasma membrane-type	
		CL9430.Contig2	−4.12692914	Down	0.00000702	0.000163392	XP_006404763.1		
		Unigene13893	−20.49563	Down	6.02E-08	0.00000216	AAF18608.2		
3	ACOX1	CL10760.Contig1	−8.01825105	Down	6.27E-11	3.53E-09	XP_006410738.1	Peroxisomal acyl-coenzyme A oxidase 1	Cell wall
4	AGD10	Unigene2844	−22.26378	Down	2.08E-08	0.000000814	XP_006410680.1	ADP-ribosylation factor GTPase-activating protein	
5	AGD13	Unigene3446	−4.082284653	Down	3.42E-18	4.05E-16	XP_006396608.1	ADP-ribosylation factor GTPase-activating protein AGD13	
6	AGL18	Unigene20308	−17.94896	Down	0.000000392	0.0000119	XP_006416569.1	Agamous-like MADS-box protein AGL18	
7	AMS	CL228.Contig1	−3.626573007	Down	0.0000214	0.000440987	XP_006409372.1	Transcription factor ABORTED MICROSPORES	Tapetum
		CL228.Contig10	−3.211951041	Down	1.49E-09	7.07E-08	XP_006300017.1		Tapetum
		CL228.Contig11	−3.981481531	Down	6.96E-15	6.28E-13			Tapetum
		CL228.Contig2	−3.888023907	Down	1.88E-12	1.3E-10	CAD54298.1		Tapetum
		CL228.Contig3	−6.49960103	Down	0.00000777	0.000178796			Tapetum
		CL228.Contig4	−3.779024655	Down	5.58E-14	4.55E-12	XP_006300017.1		Tapetum
		CL228.Contig7	−3.399639006	Down	1.8E-11	1.11E-09			Tapetum
		CL228.Contig8	−3.618863472	Down	1.7E-09	7.93E-08	CAD54298.1		Tapetum
		CL228.Contig9	−3.282505028	Down	9.96E-10	4.84E-08	XP_006300017.1		Tapetum
8	BT3	Unigene8957	−3.999145848	Down	0.000000327	0.0000102	XP_006307699.1	BTB/POZ and TAZ domain-containing protein 3	
9	CCR2	CL13286.Contig4	−2.618288933	Down	9.76E-09	0.000000404	AEK27166.1	Cinnamoyl-CoA reductase 2	Cell wall
10	ENL2	CL9725.Contig1	−12.9847855	Down	6.75E-08	0.0000024	AAR13697.1	Early nodulin-like protein 2	Tapetum
11	GALE1	CL7725.Contig1	−5.650465262	Down	0.0000241	0.000490652	XP_006391772.1	UDP-glucose 4-epimerase 1	
12	GEX1	CL6689.Contig1	−4.118156527	Down	4.93E-08	0.0000018	XP_006401451.1	Protein GAMETE EXPRESSED 1	
		CL6689.Contig2	−2.506677002	Down	0.0000292	0.000580342			
13	GUN2	CL5998.Contig1	−6.849121307	Down	6.22E-24	1.09E-21	XP_006307252.1	Endoglucanase 2	Cell wall
14	MS1	CL11348.Contig1	−3.49023522	Down	0.0000132	0.000286238	XP_006400732.1	PHD finger protein MALE STERILITY 1	Tapetum
15	MYB39	CL668.Contig2	−4.224047618	Down	0.0000012	0.0000332	NP_200422.1	Transcription factor MYB39	Tapetum
16	NAS2	Unigene9840	−10.01145026	Down	5.04E-33	1.44E-30	XP_006413176.1	Nicotianamine synthase 2	
17	ORTH2	CL8531.Contig2	−6.729731899	Down	3.32E-28	7.15E-26	XP_002886735.1	E3 ubiquitin-protein ligase ORTHRUS 2	Tapetum
		CL8531.Contig6	−6.833897659	Down	4.79E-23	7.97E-21			Tapetum
18	PAL1	Unigene4968	−2.11264418	Down	3.77E-08	0.00000142	ABC69917.1	Phenylalanine ammonia-lyase 1	
19	PAL2	Unigene23252	−3.176980409	Down	0.0000497	0.000916371	ADL09136.1	Phenylalanine ammonia-lyase 2	
20	PEAM3	Unigene26733	−2.067508491	Down	6.93E-08	0.00000246	XP_006390538.1	Phosphoethanolamine N-methyltransferase 3	
21	PGIP2	CL3211.Contig3	−4.490292387	Down	0.0000128	0.000278848	ABX46561.1	Polygalacturonase inhibitor 2	Cell wall
22	PLA2C	Unigene36790	−11.00893115	Down	3.58E-16	3.65E-14	XP_006412837.1	Phospholipase A2-gamma	
23	PLRX1	CL9725.Contig2	−11.98149635	Down	3.92E-08	0.00000146	AAR13697.1	Pollen-specific leucine-rich repeat extensin-like protein 1	Tapetum
24	PME5	Unigene14803	−24.662	Down	2.79E-71	3.17E-68	ABC25451.1	Pectinesterase 5	Cell wall
		Unigene37636	−24.55937	Down	3.24E-70	3.45E-67	XP_006397894.1		Cell wall
25	RBG7	Unigene958	−1.955925101	Down	0.00000713	0.000165484	NP_179760.1	Glycine-rich RNA-binding protein 7	Cell wall
26	RGP1	CL5431.Contig2	−3.796328409	Down	2.54E-20	3.52E-18	XP_006408433.1	UDP-arabinose mutase 1	Cell wall
27	TI10A	CL3440.Contig3	−1.838249753	Down	0.00000272	0.0000696	XP_006416530.1	jasmonate-zim-domain protein 1	
28	TMK1	Unigene30627	−17.95435	Down	0.00000625	0.000147115	XP_002867400.1	Probable receptor protein kinase	Cell wall
29	TSM1	Unigene12653	−4.917563543	Down	0.0000129	0.000281326	NP_564916.2	Tapetum-specific methyltransferase 1	Tapetum
		Unigene4469	−7.200986245	Down	0.00000372	0.0000924	XP_002888647.1		Tapetum
30	ZAT5	CL12866.Contig2	−17.40146	Down	0.0000171	0.000360205	NP_187658.1	Zinc finger protein	Cell wall
		CL801.Contig1	−6.072505988	Down	6.22E-16	6.25E-14	XP_006398898.1		Cell wall
		CL801.Contig4	−6.376284185	Down	0.000000604	0.0000178			Cell wall
		CL801.Contig5	−17.48876	Down	0.0000089	0.000202113	XP_002871091.1		Cell wall
31	BT2	Unigene16869	4.243001335	Up	0.0000312	0.000613786	XP_004975990.1	BTB/POZ and TAZ domain-containing protein 2	
32	SCC12	CL8357.Contig1	4.33729296	Up	0.00000218	0.0000571	XP_006405422.1	Sister chromatid cohesion 1 protein 2	
33	TCMO	Unigene3794	4.003339344	Up	0.0000226	0.000461499	XP_006410152.1	Trans-cinnamate 4-monooxygenase	Cell wall
34	VAL2	CL7862.Contig2	6.699055286	Up	2.06E-15	1.96E-13	NP_194929.2	B3 domain-containing transcription repressor	Cell wall
		CL7862.Contig7	8.577233976	Up	1.62E-19	2.14E-17			Cell wall
35	XPO1	CL3131.Contig8	17.77931	Up	0.00000304	0.0000768	XP_006400241.1	Exportin1 (XPO1) like protein	

### Differentially expressed transcription factor genes

**Fig. 4:**
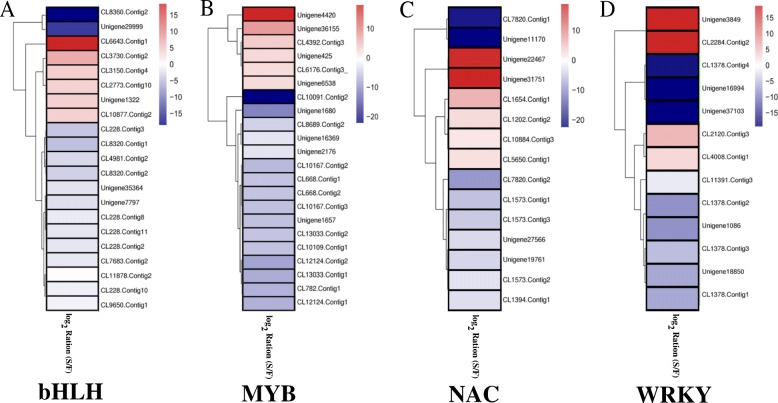
Heat map analysis of bHLH (**a**), MYB (**b**), NAC (**c**) and WRKY (**d**) transcription factors. The expression levels shown are based on RPKM data. The color key represents the value of log2(S/F). Red represents high expression, and blue represents low expression. Each row represents a DEG

In the anther and pollen development processes, transcription factors are generally thought to be important regulators. To identify differentially expressed transcription factors, all of the DEGs were annotated. In this study, 131 transcription factors (182 DEGs) were found, including 128 down- and 54 upregulated DEGs (Additional file 9: Table S7). Among these transcription factors, 27 up- and 8 downregulated DEGs were specific to fertile and sterile buds, respectively. In addition, 13 DEGs were associated with 8 *WRKY* transcription factor genes, and *WRKY19* (Unigene3849, CL2284.Contig2, CL2120.Contig3) and *WRKY32* (CL4008.Contig1) were upregulated in only sterile buds. Fifteen DEGs were identified with 10 *NAC* transcription factor genes, and 6 of them were highly expressed in sterile buds. In the bHLH and MYB transcription factor families, a total of 43 DEGs were associated with 16 *bHLH* and 13 *MYB* transcription factors, and 10 *bHLHs* (15 DEGs) and 8 *MYBs* (16 DEGs) were downregulated in sterile buds, respectively (Fig. 4, Table 3). These differentially expressed transcription factors might result in abortion of pollen development in Wucai.

**Table 3:** Annotation and relative expression of differentially expressed bHLH, MYB, NAC and WRKY transcription factor genes in Wucai

#	Gene ID	Annotation	Base Mean F	Base Mean S	log2 Ratio (S/F)	Up-Down Regulation (S/F)	*P*-value	FDR	Nr/Nt-ID
bHLH									
1	CL228.Contig10	AMS	409.1749872	44.15861763	−3.211951041	Down	1.49E-09	7.07E-08	XP_006300017.1
2	CL228.Contig2		846.3264726	57.16446004	−3.888023907	Down	1.88E-12	1.3E-10	CAD54298.1
3	CL228.Contig3		22.71136886	0.250996948	−6.49960103	Down	0.00000777	0.000178796	CAD54298.1
4	CL228.Contig11		929.7544629	58.86035859	−3.981481531	Down	6.96411E-15	6.28153E-13	XP_006300017.1
5	CL228.Contig8		1219.003283	99.22438599	−3.618863472	Down	1.7E-09	7.93E-08	CAD54298.1
6	CL9650.Contig1	BH010	68.59317113	8.284434373	−3.04958985	Down	0.00000726	0.000168143	XP_006294062.1
7	CL4981.Contig2	BH055	20.42367727	0.570787056	−5.161146216	Down	0.0000481	0.000890179	XP_006417224.1
8	CL11878.Contig2	BH069	273.2838968	71.45109769	−1.935372371	Down	0.0000331	0.000647096	XP_006412645.1
9	Unigene35364	BH089	1343.847101	69.02695614	−4.283065324	Down	2.55E-23	4.31E-21	XP_002892324.1
10	CL8320.Contig1	BH091	354.1856344	2.682361084	−7.04485843	Down	7.68E-30	1.83E-27	XP_006410252.1
11	CL8320.Contig2		234.632662	4.474145004	−5.712648033	Down	1.03E-12	7.33E-11	XP_006410252.1
12	Unigene7797	BH095	53.40631948	2.362570976	−4.498580887	Down	1.08E-08	0.000000444	XP_006393216.1
13	CL8360.Contig2	BH100	474.3340898	0.0001	−22.17747	Down	0.000000143	0.00000478	XP_006411389.1
14	Unigene29999	BH126	18.09145412	0.0001	−17.46495	Down	0.0000168	0.0003553	XM_002869626.1
15	CL7683.Contig2	BH129	75.39194436	5.398710046	−3.80372374	Down	2.68E-08	0.00000103	XP_006411590.1
16	Unigene1322	BH075	637.9841813	2792.211601	2.129815719	Up	4.23E-08	0.00000157	XP_006415858.1
17	CL6643.Contig1	BH077	0.0001	26.3978698	18.01006	Up	0.00000272	0.0000696	ACB30989.1
18	CL3150.Contig4	BH104	47.90423927	240.6065811	2.328450867	Up	0.000000366	0.0000112	XP_002870316.1
19	CL3730.Contig2	BH135	1.485983153	67.46448335	5.504638531	Up	2.93E-11	1.74E-09	XP_006395335.1
20	CL10877.Contig2	BH149	46.40107667	203.9150311	2.135737938	Up	0.00000403	0.000099	XP_002866229.1
21	CL2773.Contig10	LHWL3	60.30986683	235.7158239	1.966582657	Up	0.0000344	0.000666052	NP_180686.2
MYB									
1	CL13033.Contig1	MY101	462.3621775	7.375614264	−5.970116369	Down	1.89E-29	4.38E-27	AF411970_1
2	CL13033.Contig2		552.9666991	27.31999076	−4.3391637	Down	6.99E-21	1E-18	AF411970_1
3	CL10091.Contig2	GAM1	37.53982746	0.0001	−18.51806	Down	0.0000439	0.000821047	NP_191605.1
4	Unigene16369	MY104	311.4872214	83.66750916	−1.896433588	Down	0.0000341	0.000661013	XP_006408602.1
5	CL10109.Contig1	MY1R1	36.53415274	1.717675964	−4.410715944	Down	0.00000123	0.000034	XP_006297882.1
6	CL782.Contig1		42.56716483	0.959370325	−5.471509378	Down	0.000000107	0.00000366	XP_006394486.1
7	Unigene1680		2848.722771	6.101768627	−8.866872039	Down	1.03E-21	1.55E-19	XP_006394486.1
8	CL668.Contig1	MYB38	752.2749053	43.38701927	−4.115924585	Down	0.00000243	0.0000631	NP_200422.1
9	CL668.Contig2	MYB39	72.94632466	3.903357947	−4.224047618	Down	0.0000012	0.0000332	NP_200422.1
10	CL8689.Contig2	MYB44	85.46534877	8.667702846	−3.30161801	Down	5.52E-08	0.000002	XP_006391000.1
11	CL10167.Contig2		55.89646529	1.738835419	−5.006563761	Down	7.71E-10	0.000000038	NP_173237.1
12	CL10167.Contig3		22.38440326	0.959370325	−4.544262234	Down	0.0000475	0.000881123	NP_173237.2
13	Unigene1657		35.26630754	1.508997926	−4.546627802	Down	0.000000811	0.0000233	AAC83612.1
14	CL12124.Contig1	MYB86	174.6674392	3.599412499	−5.600705435	Down	4.39E-20	5.99E-18	XP_006416547.1
15	CL12124.Contig2		78.19330659	0.848258255	−6.526397743	Down	1.18E-14	1.04E-12	XP_006416547.1
16	Unigene2176		321.6680886	80.21008064	−2.003717351	Down	0.00000411	0.000100816	XP_006407153.1
17	CL6176.Contig3	MB3R1	10.12504634	76.35690834	2.914830179	Up	0.00000206	0.0000544	XP_006399657.1
18	CL4392.Contig3	MYB28	8.25033752	156.5456644	4.245986602	Up	4.33E-10	0.000000022	ACR48179.1
19	Unigene425		15.57701305	144.8393522	3.216963107	Up	0.00000604	0.000142804	AFX96283.1
20	Unigene6538	MYB29	11.11907995	85.93982834	2.950289479	Up	0.00000586	0.00013881	ACR48181.1
21	Unigene36155	MYB48	0.742994303	262.1628707	8.462896512	Up	8.61E-14	6.87E-12	AFW74899.1
22	Unigene4420	MYB59	0.0001	35.65819104	18.44387	Up	0.00000027	0.00000848	NP_851226.1
NAC									
1	Unigene27566	NAC06	40.52594362	3.321941301	−3.608747293	Down	0.00000538	0.00012842	XP_006411314.1
2	CL1573.Contig1	NAC18	39.29102704	0.821784004	−5.579296819	Down	2.33E-08	0.000000909	ACN62416.1
3	CL1573.Contig2		872.2221035	131.168863	−2.733270259	Down	2.46E-11	1.48E-09	XP_006406961.1
4	CL1573.Contig3		505.1184416	17.11619779	−4.883187554	Down	1.48E-22	2.38E-20	XP_002882948.1
5	CL7820.Contig1	NAC25	114.7087842	0.0001	−20.12954	Down	3.58E-22	5.55E-20	AAB71483.1
6	CL7820.Contig2		4465.490482	7.822460828	−9.156980403	Down	4.64E-38	1.66E-35	NP_564771.1
7	Unigene11170		572.1354143	0.0001	−22.44793	Down	1.44E-30	3.53E-28	AAM65392.1
8	CL1394.Contig1	NAC69	376.7708871	42.48727739	−3.14858469	Down	3.24E-11	1.91E-09	XP_006286761.1
9	Unigene19761	NAC78	63.37497158	4.479459799	−3.822516529	Down	0.000000543	0.0000162	XP_006399623.1
10	CL1654.Contig1		1.494589233	109.339016	6.192915453	Up	2.7E-16	2.78E-14	XP_006398899.1
11	Unigene22467	NAC18	0.0001	26.72752603	18.02797	Up	0.000000402	0.0000122	DAA48262.1
12	CL10884.Contig3	NAC29	323.0703998	1086.313175	1.7495196	Up	0.00000775	0.00017832	XP_006399904.1
13	CL5650.Contig1	NAC31	134.1180934	613.9217676	2.194550945	Up	7.76E-08	0.00000273	XP_006412943.1
14	CL1202.Contig2	NAC42	17.5549512	122.8209502	2.806606779	Up	0.000000194	0.00000629	XP_006411573.1
15	Unigene31751	NAMB2	0.0001	45.35108693	18.79078	Up	7.94E-11	4.41E-09	XP_006292493.1
WRKY									
1	CL11391.Contig3	WRKY2	352.3112665	104.7828505	−1.749447996	Down	0.000031	0.000611096	ACQ76801.1
2	Unigene18850	WRKY9	64.21010222	0.618420761	−6.698067731	Down	0.00000303	0.0000765	AHB33821.1
3	Unigene16994	WRKY10	62.20024848	0.0001	−19.24656	Down	5.45E-13	3.98E-11	XP_006392618.1
4	CL1378.Contig1	WRKY58	239.5168401	2.26198877	−6.726391516	Down	5.4E-17	5.87E-15	AHB33854.1
5	CL1378.Contig2		287.5616426	0.890577165	−8.334914915	Down	1.47683E-32	4.10153E-30	XP_006408460.1
6	CL1378.Contig3		44.85334435	1.210367273	−5.21169877	Down	7.66E-09	0.000000323	AHB33854.2
7	CL1378.Contig4		21.15655342	0.0001	−17.69075	Down	0.00000189	0.0000501	NP_683519.2
8	Unigene1086	WRKY64	198.2663101	0.618420761	−8.324635083	Down	1.61E-09	7.54E-08	AHB33859.1
9	Unigene37103	WRKY66	65.05180056	0.0001	−19.31123	Down	2.76E-15	2.59E-13	AHB33861.1
10	Unigene3849	WRKY19	0.0001	42.24559068	18.68844	Up	0.00000337	0.0000845	XP_002869985.1
11	CL2120.Contig3		2.211765296	127.96661	5.85442531	Up	6.61E-16	6.61E-14	XP_006399450.1
12	CL2284.Contig2		0.0001	44.62232571	18.76741	Up	0.00000015	0.00000499	ACP30636.1
13	CL4008.Contig1	WRKY32	6.738547034	57.69680762	3.09798204	Up	0.00000546	0.000130266	AHB33838.1

### Real-time qPCR validation of gene expression patterns

**Fig. 5:**
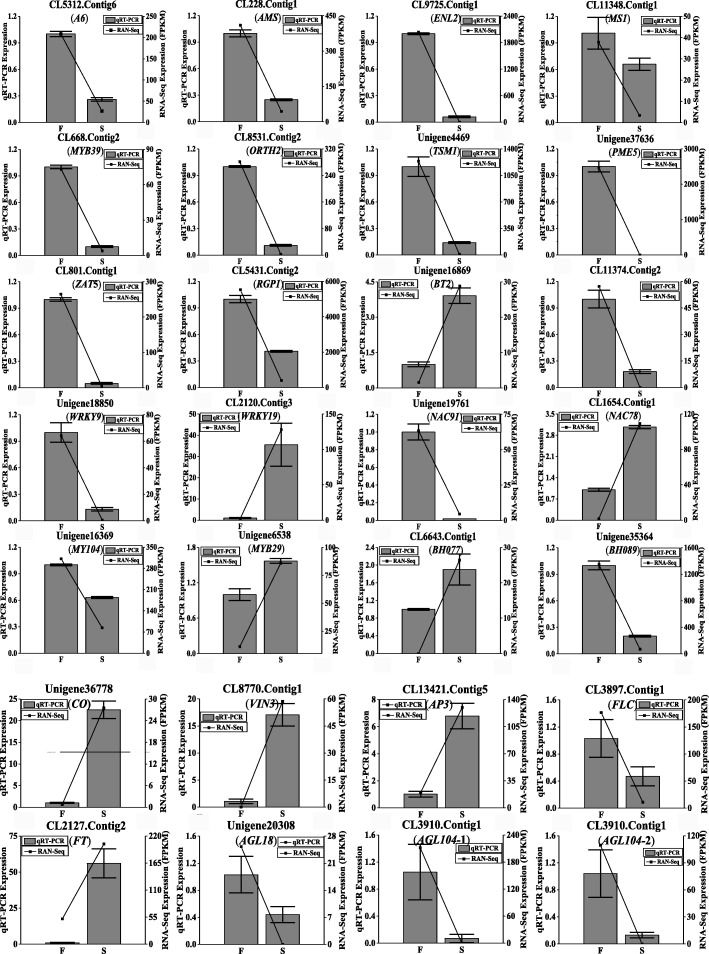
qRT-PCR verification of differentially expressed unigenes. S means sterile sample, and F means fertile sample. Relative expression levels were calculated using *Actin* as an internal control

To validate the results of RNA-Seq, 28 DEGs, including 11 genes annotated to anther and pollen development, 8 transcription factor genes, 8 flowering genes and one gene with unknown function were subjected to verification using qRT-PCR. The results of this experiment are shown in Fig. 5. Among these genes, 19 genes were downregulated in sterile buds, including 7 tapetum-specific genes (*A6*, *AMS*, *ENL2*, *MS1*, *MYB39*, *ORTH2* and *TSM1*), 3 pollen cell wall formation genes (*PME5*, *ZAT5*, *RGP1*), 4 transcription factor genes (*WRKY9*, *NAC91*, *MY104*, *BH089*), 4 flowering genes (*FLC*, *AGL18*, *AGL104–1* and *AGL104–2*) and one gene with unknown function (CL11374.Contig2). All of the 28 DEGs exhibited the same tendency between the RNA-Seq analysis and qRT-PCR results, which suggested that our transcriptome analysis was accurate and reliable.

**Fig. 6:**
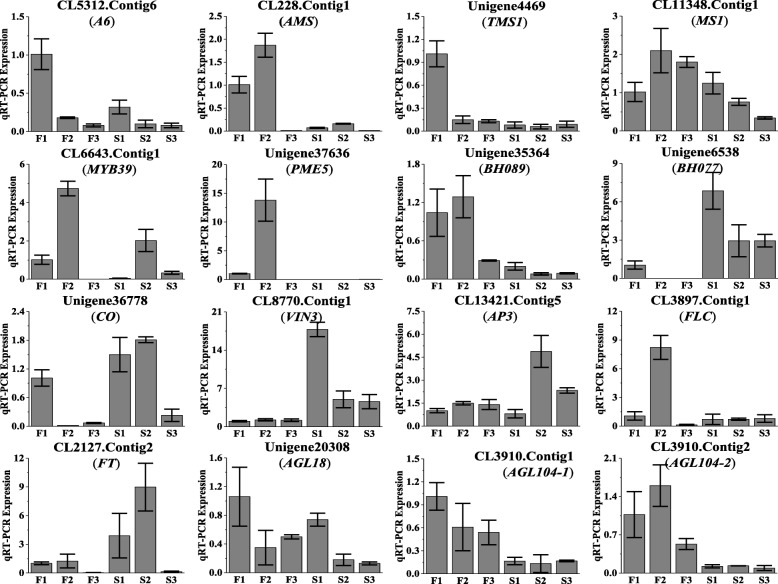
Expression of anther and pollen development related genes at different stages using qRT-PCR. S means sterile sample, and F means fertile sample. 1–3 indicate the pollen meiosis stage (bud sizes 0.5–1.5 mm), tetrad stage (1.5–3.0 mm) and uninucleate microspore stage (3.0–4.5 mm) of anther and pollen development, respectively. Relative expression levels were calculated using *Actin* as an internal control

To further determine the expression pattern of key genes in the anther and pollen development, 2 transcription factors and 6 tapetum and pollen cell wall development genes were selected from the above for qRT-PCR assay (Fig. 6). Among these genes, the pollen cell wall formation gene *PME5* (Unigene37636) was highly and specifically expressed in the fertile buds at the tetrad stage. The five tapetum development genes and one transcription factor (*BH089*) were highly expressed at the meiosis or tetrad stage in the fertile buds, and all of them shoed low abundance in sterile buds. The other transcription factor, *BH077*, was highly expressed at meiosis in sterile buds. These results further confirmed the reliability of the RNA-Seq data.

**Fig. 7:**
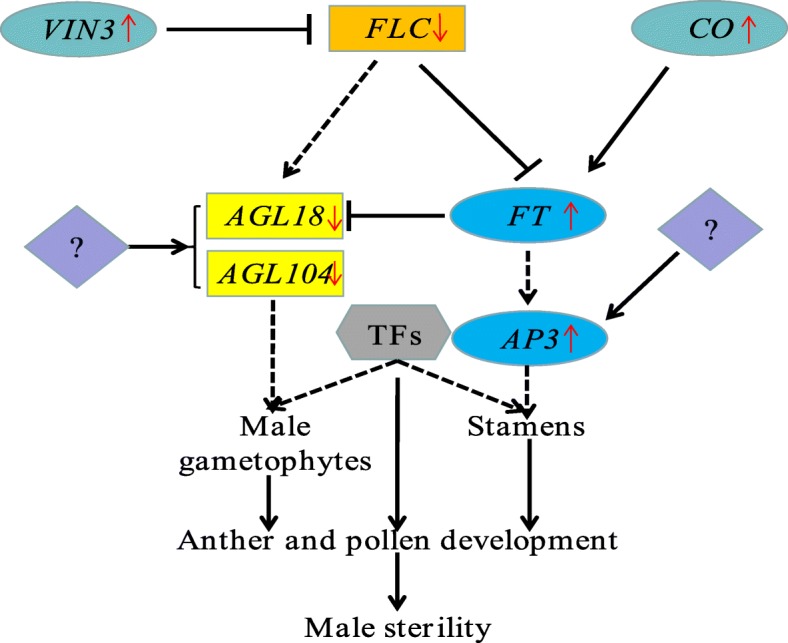
The mechanism of FLC affects fertility in Wucai. *Black arrows* indicate promotion, and inverted *T bars* indicate repression. *Dashed lines* indicate that further research is needed. Question marks indicate unknown genes. *Red arrows* indicate up- or downregulation. *AGL18* (Unigene20308), *AGAMOUS LIKE 18*; *AGL104* (CL3910.Contig1, CL3910.Contig2), *AGAMOUS LIKE 104*; *AP3* (CL13421.Contig5), *APETALA 3*; *CO* (Unigene36778) *CONSTANS*; *FLC* (CL3897.Contig1), *Flowering Locus C*; *FT* (CL2127.Contig2), *Flowering Locus T*; *VIN3* (CL8770.Contig1), *VERNALIZATION 3*; TFs, transcription factors

In addition, 8 flowering genes were also examined in this study (Fig. 6). Among these genes, *CO*, *AP3* and *FT* were highly expressed at the tetrad stage, and *VIN3* was highly expressed in the meiosis period in sterile buds. *FLC*, *AGL18*, *AGL104–1* and *AGL104-2* were highly expressed at the meiosis or tetrad stage in fertile buds. The abnormal expression of these genes might influence the development of male gametophytes and stamens, leading to male sterility (Fig. 7).

## Discussion

In higher plants, male sterility is a common phenotypic trait in which the abortion of stamens occurs and plants fail to produce functional anthers, pollen or male gametes under typical natural conditions [20, 24]. As the male reproductive organ, stamens play an important role in plant inheritance [5]. In this present study, morphological comparisons were performed between fertile and sterile lines of Wucai (Fig. 1a-h), and there was no difference between them except the stamens, which had shorter filaments and aborted anthers in the sterile flowers (Fig. 1g-h). A cytological examination was further carried out to evaluate the differences in pollen development between the fertile and sterile lines, and we observed that anther abortion occurred consistently in the sterile line, in which the tapetum developed abnormally and the microspore began to degrade after the meiotic stage (Fig. 1i-l). These results were consistent with those of Liu et al. [7] and Zhou et al. [5] and suggested that the abnormal development of the tapetal cells and microspores led to pollen and anther abortion.

To better identify the genes associated with pollen abortion in this CMS line of Wucai, a comprehensive analysis of transcript profiles between fertile buds and sterile buds was performed using RNA sequencing technology, which could detect low abundance transcripts and provide new insights into male sterility through global investigation of gene expression changes [5, 25]. A total of 105,543,483 clean reads and 117,332 contigs were obtained based on the RNA-Seq data, and 980 upregulated (1.21%) and 3450 downregulated (4.27%) DEGs out of 80,851 unigenes were identified based on their gene expression levels (Fig. 2; Table 1; Additional file 4: Table S3). These results indicated that changes in the expression of a large number of related genes could cause male sterility in Wucai, though the development of anther and pollen is a complicated process and involves numerous genes.

In the KEGG enrichment results, 4430 DEGs were classified into 119 metabolic pathways (Fig. 3, Additional file 7: Table S5), and these pathways might encompass all the biological pathways in anther development [26]. Among these pathways, starch and sucrose metabolism provides energy and carbon for anther development, and starch and sucrose are accumulated as energy reserves for pollen maturation [4, 27]. In our research, out of 124 DEGs involved in this pathway, 38 DEGs were expressed in only fertile buds and 2 DEGs (Unigene23056 and Unigene11909) were expressed in only sterile buds (Additional file 8: Table S6). The specific expression of these genes might lead to disturbances in the metabolism of starch and sucrose and the processing of energy reserves, which could suppress pollen development and ultimately lead to male sterility [7, 28]. This finding was consistent with those of previous works [4, 19, 29, 30].

In addition to the metabolic pathways, many key genes have been identified for pollen and anther development in *Arabidopsis* [31] and *Brassica* [9, 20, 32]. It is important to note that we identified 35 anther and pollen development related genes (53 unigenes) that have homologs in *Arabidopsis* and *Brassica*, and most of them were downregulated and associated with the development of the tapetum and pollen cell wall (Table 2). Among these 53 unigenes, 9 DEGs (6 genes: *ACA2*, *AGD10*, *AGL18, PME5*, *TMK1* and *ZAT5*) were expressed in only fertile buds (log_2_ Ratio(S/F) > 17), which might offer new insights into the mechanisms of CMS regulation in Wucai. *ACA2* encodes a Calcium-transporting ATPase 2 (plasma membrane-type), which regulates the Ca^2+^-mediated signaling pathway during pollen development [33, 34]. The nonexpression of this gene in sterile buds might disrupt the Ca^2+^ balance in the pollen mother cell. However, interestingly, *AGD10* might be involved in root development as an ARF-GAP protein [35–37], and *AGL18*, encoding a MADS-box protein, has been reported as a flowering-inhibiting factor [38]. The functions of these genes in pollen development have not yet been reported and need further investigation. The other three genes (*PME5*, *ZAT5*, *TMK1*) and *ACOX1*, *CCR2*, *GUN2*, *PGIP2*, *RBG7*, *RGP1*, *TCMO*, *VAL2* were involved in pollen cell wall formation, modification and organization (Table 2), in which critical chemical changes could lead to pollen abortion [5].

It has been reported that some constituents of the pollen wall are secreted from tapetal cells [39, 40]. Abnormal (early or delayed) tapetal cell degeneration can result in male sterility [5]. In conjunction with our cytological observations of Wucai buds (Fig. 1i-p), several genes related to tapetum development were revealed (Table 2). As a basic helix-loop-helix (bHLH) protein, *AMS* is required for tapetal cell biosynthesis, postmeiotic microspore and pollen wall formation, and tapetum programmed cell death (PCD) by directly regulating target genes involved in these biological pathways [41–43]. *MS1* encodes a transcription factor of the PHD finger family and is specifically expressed in microsporocytes [44]. *A6*, a tapetum-specific protein secreted by the tapetal cells, displays similarity to β-1,3-glucanases, which degrade callose during pollen development [45]. *TSM1* encodes a cation-dependent CCoAOMT-like protein involved in phenylpropanoid polyamine conjugate biosynthesis and has a function in stamen/pollen development [46, 47]. Downregulated expression of these genes could result in degeneration of the tapetum, eventually leading to abortion.

The regulation of transcription is a fundamental process in all living organisms [48]. Transcription factors can regulate multiple related downstream genes, which are essential components of the cellular machinery and play key roles in plant growth and development [49]. In the present study, 131 transcription factors (182 DEGs) were found (Additional file 9: Table S7). Among these transcription factors, the top four largest families were *bHLH* (16), *MYB* (13), *NAC* (10), and *WRKY* (8) (Table 3). The bHLH proteins, which bind as dimers to specific DNA target sites, are a superfamily of transcription factors, and several of them are critical for tapetal PCD and pollen development [41]. *MYB* transcription factors are also known to be required for anther and aleurone layer development, callose dissolution, and exine formation [19, 50, 51]. *NAC* and *WRKY* transcription factors consist of a large gene family involved in a wide range of biological processes [48, 50], and some of them participate in pollen development (*WRKY2*, *WRKY27*; *GPC*, *NST1*) [48, 52–54]. Research over the past several years has demonstrated that changes in the expression of these transcription factors often cause male sterility [5, 19].

In addition, *FLOWERING LOCUS C* (*FLC*), which encodes a MADS-box transcription factor and functions as a repressor of flowering [55], was noted in our comparative analysis (CL3897.Contig1; Additional file 9: Table S7). It has been reported that overexpression of this gene from *B. campestris* could affect fertility by the GA pathway in *Arabidopsis* [56]. However, in our study, we found that the *FLC* gene was downregulated in sterile buds, and several identified genes involved in stamens (*AP3*) [57] and the male gametophyte (*AGL18* and *AGL104*) [58, 59] (Fig. 6; Additional file 10: Table S8) were downstream targets of *FLC* (Fig. 7). Among these genes, *AGL18* and *AGL104* showed low expression in fertile buds (Fig. 6; Additional file 10: Table S8). We speculated that the downregulation of *FLC*, which is associated with male infertility, might influence the expression of key genes in anther and pollen development, along with other fertility related genes (Fig. 7). This hypothesis must be further verified.

Taken together, the present investigation of the transcriptome could increase our knowledge and understanding of the molecular mechanisms of male sterile in Wucai and provide numerous candidate genes that can be verified through transgenic technology in future.

## Conclusions

In this study, a comparative transcriptome analysis of sterile and fertile buds from Wucai was performed through an Illumina sequencing approach, and the different biological processes and genes that regulated anther and pollen development were analyzed using comparative analysis. As a result, a total of 4430 DEGs, 174 novel genes, 35 anther and pollen development related genes, and 47 transcription factors (the top four largest families) were revealed. The RNA-Seq analysis was further confirmed through qRT-PCR. Based on the functional annotation and expression patterns, it was concluded that the occurrence of male sterility is probably related to the functional and metabolic abnormalities of these candidate DEGs in Wucai. These transcriptome data will be important to serve as a reference and provide insights for future elucidation of male sterility in Wucai.

## Materials and Methods

### Plant materials

Buds from near-isogenic lines of Wucai, CMS line 12-14A and its maintainer line 12-14B (Fig. 1a and b), were used as the plant materials in this study. Backcrossed continuously for over ten generations, the male sterile line 12-14A of Wucai was generated from a CMS line of nonheading Chinese cabbage. The sterile line 12-14A and its maintainer line, 12-14B, were planted in the vegetable breeding fields of Anhui Agricultural University (Hefei, Anhui Province, China; longitude 117°14′E, latitude 31°52’N) from October until April of the following year.

### Morphological and cytological observations

At the full-bloom stage, the flower structures of the CMS and fertile lines were observed using a Canon EOS550D digital camera (Canon, Japan), and the images from petals and stamens were captured with an Olympus SZX10 stereomicroscope (Olympus, Japan). The sections of flower buds from the CMS and fertile plants were obtained following the method described by Peng et al. [60]. The semithin sections were observed and photographed using an Olympus BX61 light microscope (Olympus, Japan) equipped with a Mshot MD30 camera (Olympus, Japan).

### RNA extraction and Illumina sequencing

In this experiment, the buds of the sterile or fertile lines were collected from three different plants, respectively. According to the methods of Huang et al. [25], total RNA was isolated from the mixed bud samples using the TRIzol Reagent Kit (Invitrogen, USA) and purified using the Dynabeads® mRNA Purification Kit (Ambion, USA). The isolated RNA samples were sent to 1GENE Technology Co., Ltd. (Hangzhou, China; http://www.1gene.com.cn/) for Illumina sequencing (Illumina MiSeq platform) and unigene annotation. The raw transcriptome data of six samples from three biological replicates of sterile or fertile lines were deposited in the NCBI Short Read Archive (SRA, accession number: SRP145484).

### De novo assembly and functional annotation analysis

The paired-end clean reads of each sample were de novo assembled into contigs using Trinity (http://trinityrnaseq.sourseforge.net), and the nonredundant unigenes were further obtained with the TGI Clustering tools [61]. Among these unigenes, there were several unigenes with a high degree of similarity (more than 70%) in the same cluster (starting with CL, followed by the gene family’s number); the rest were singletons (starting with Unigene), which had low similarity (less than 70%) or no similarity and could not be clustered with each other. Then, functional annotation of the unigenes was performed using BLASTX alignment (E-value<1e-5) in the nonredundant (nr), Swiss-Prot, and COG databases. With the nr annotations, the Gene Ontology (GO) annotations of the unigenes were obtained through the Blast2GO program [62], and GO functional classification was carried out with the WEGO software [63]. The KEGG pathway annotation was performed using a BLAST search against the KEGG database (KEGG, http://www.genome.jp/kegg/).

### Differentially expressed gene (DEG) identification

Reads per kilobase per million reads (RPKM) was adopted to compare the differences in unigene expression between the sterile and fertile lines. The DEGs were identified by a false discovery rate (FDR) ≤0.001 and an absolute value of log_2_ ratio ≥ 1 (ratio = the fold change of differential expression) [13]. The DEGs were used for GO and KEGG enrichment analyses according to the method described by An et al. [14] and Liu et al. [13].

### Quantitative real-time PCR verification

RNA was isolated from different samples as described above. The DNase-treated RNA (1 mg) was reverse transcribed to cDNA using the PrimeScript™ RT Reagent Kit (TaKaRa, Japan). Quantitative real-time PCR was then performed with the SYBR® *Premix Ex Taq*™ II Kit (TaKaRa, Japan). The specific primers designed based on the selected DEG sequences are listed in Additional file 11: Table S9. PCR amplification was performed in the Bio-Rad CFX96 instrument according to the manufacturer’s instructions. Data normalization was carried out using the expression levels of *Actin* as the internal control. Three biological repeats for each sample and three technical replicates for each gene were performed, and the relative expression level was calculated as 2^-ΔΔCt^.

## Additional files

Additional file 1: **Table S1.** Summary of de novo transcriptome assembly. (XLS 18 kb)

Additional file 2: **Table S2.** Statistics of annotation results. (XLS 18 kb)

Additional file 3: **Figure S1.** Characteristics of homology search of Illumina sequences against the NR database. (DOC 505 kb)

Additional file 4: **Table S3.** All DEGs between sterile and fertile buds. (XLS 5575 kb)

Additional file 5: **Table S4.** Novel genes. (XLS 50 kb)

Additional file 6: **Figure S2.** Gene Ontology (GO) assignment. of DEGs. (DOC 190 kb)

Additional file 7: **Table S5.** Statistics of DEGs matched in KEGG pathways. (XLS 41 kb)

Additional file 8: **Table S6.** DEGs associated with starch and sucrose metabolism. (XLS 46 kb)

Additional file 9: **Table S7.** Annotation and relative expression of differentially expressed transcription factors in Wucai. (XLS 60 kb)

Additional file 10: **Table S8.** Flowering genes identified from the DEGs. (XLS 21 kb)

Additional file 11: **Table S9.** Primers used in this study. (XLS 26 kb)

## Abbreviations

CMS: Cytoplasmic male sterility
DEGs: Differentially expressed genes
FDR: False discovery rate
FLC: FLOWERING LOCUS C
GO: Gene Ontology
KEGG: Kyoto Encyclopedia of Genes and Genomes
PCD: Programmed cell death
RNA-Seq: RNA sequencing technology
RPKM: Reads per kilobase per million reads
SRA: Short Read Archive
