# Supplementary material for: Comparative Transcriptome Analysis between Fertile and CMS Flower Buds in Wucai (Brassica campestris L.)
Source: BMC Genomics. 2018 Dec 12;19:908. doi: 10.1186/s12864-018-5331-4 (PMC6292171; doi:10.1186/s12864-018-5331-4)
Supplement: Supplementary file 3 — Figure S1. Characteristics of homology search of Illumina sequences against the NR database. (DOC 505 kb) [file 12864_2018_5331_MOESM3_ESM.doc]

**Additional file 3: Figure S1. Characteristics of homology search of Illumina sequences against the NR database.**

A, E-value distribution of BLAST hits for each unique sequence with a cut-off E-value of 1.0E-5. B, Similarity distribution of the top BLAST hits for each sequence. C, Species distribution is shown as a percentage of the total homologous sequences with an E-value of at least 1.0E-5. We used the first hit of each sequence for analysis.
