# Supplementary material for: Comparative Transcriptome Analysis between Fertile and CMS Flower Buds in Wucai (Brassica campestris L.)
Source: BMC Genomics. 2018 Dec 12;19:908. doi: 10.1186/s12864-018-5331-4 (PMC6292171; doi:10.1186/s12864-018-5331-4)
Supplement: Supplementary file 6 — Figure S2. Gene Ontology (GO) assignment. of DEGs. (DOC 190 kb) [file 12864_2018_5331_MOESM6_ESM.doc]

**Additional file 6: Figure S2. Gene Ontology (GO) assignment of DEGs.**

The X-axis indicates the subcategories; the left Y-axis indicates the percentage of a subcategory of genes in that category, and the right Y-axis indicates the number of unigenes in a subcategory. Red represents significantly upregulated genes, and blue represents significantly downregulated genes.
